# Disparate Molecular Properties of Two Hypertrophic Cardiomyopathy-Associated cMyBP-C Mutants Reveal Distinct Pathogenic Mechanisms Beyond Haploinsufficiency

**DOI:** 10.3390/biomedicines13051010

**Published:** 2025-04-22

**Authors:** Angelos Thanassoulas, Emna Riguene, Maria Theodoridou, Laila Barrak, Hamad Almaraghi, Mohammed Hussain, Sahar Isa Da’as, Mohamed A. Elrayess, F. Anthony Lai, Michail Nomikos

**Affiliations:** 1 College of Medicine, QU Health, Qatar University, Doha P.O. Box 2713, Qatarlaila.anbari.barrak@gmail.com (L.B.);; 2 Biomedical Research Center, QU Health, Qatar University, Doha P.O. Box 2713, Qatar; 3 Research Department, Sidra Medicine, Doha P.O. Box 26999, Qatar; sdaas@sidra.org; 4 College of Health and Life Sciences, Hamad Bin Khalifa University, Doha P.O. Box 34110, Qatar

**Keywords:** hypertrophic cardiomyopathy, HCM, *MyBPC3*, cMyBP-C, cardiac disease

## Abstract

**Background/Objectives**: Hypertrophic cardiomyopathy (HCM) is a common genetic cardiac disorder marked by abnormal thickening of the left ventricular myocardium, often leading to arrhythmias and heart failure. Mutations in sarcomeric protein genes, particularly *MYBPC3*, which encodes cardiac myosin-binding protein C (cMyBP-C), are major contributors to HCM pathogenesis. This study aims to investigate the structural and functional effects of two HCM-associated missense mutations, p.S236G and p.E334K, located within the C0–C2 domains of cMyBP-C. **Methods**: Following in silico analysis, a bacterial expression system was applied, enabling the discrete C0–C2 domains of wild-type (cMyBP-C^WT^) and mutant (cMyBP-C^S236G^ and cMyBP-C^E334K^) cMyBP-C proteins to be expressed and purified as recombinant proteins. Structural and stability changes were assessed using circular dichroism (CD), differential scanning calorimetry (DSC), and chemical denaturation assays. Functional impact on actin binding was also evaluated in vitro. **Results**: CD analysis revealed altered secondary structure in both mutants compared to the wild-type protein. Thermal and chemical stability assays indicated increased stability in the cMyBP-C^E334K^ mutant, suggesting that it exhibits a more rigid conformation. This increased rigidity corresponded with a significant reduction in the actin-binding affinity relative to the wild-type protein. **Conclusions**: Our findings demonstrate specific detrimental effects of the p.E334K mutation and underscore the importance of understanding the structural and functional consequences of HCM-associated mutations to assist the development of targeted therapeutic strategies.

## 1. Introduction

Hypertrophic cardiomyopathy (HCM; OMIM#192600) is a common hereditary disease, with an estimated phenotypic prevalence of 1 in 500 and a potential genotypic prevalence of 1 in 200 [1]. The main characteristic of HCM is the asymmetrical thickening of the left ventricle (LV) walls, occurring in the absence of elevated blood pressure or other intrinsic cardiovascular or systemic disorders [2]. Thickening of the walls can hinder diastolic function, obstruct the outflow tract of the left ventricle, and cause arrhythmias, such as atrial fibrillation (AF) or ventricular tachycardia (VT) [3,4]. These cardiac problems lead to significant cardiovascular complications, collectively described as HCM.

HCM has a wide range of clinical features. Patients affected by HCM may experience a variety of symptoms, including fatigue, palpitations, exertional dyspnea, fatigue, atypical chest pain, and sudden cardiac death (SCD), resulting from ventricular diastolic dysfunction and cardiac arrhythmias as primary pathophysiological conditions [2,5]. In most cases, HCM manifests with a relatively benign phenotype. However, it is also an important cause of sudden cardiac death, particularly in teenagers and young adults [2], due to the presence of certain mutations, especially in genes that encode some structural and regulatory proteins within cardiomyocytes, which can eventually result in cardiomyopathy [6].

HCM is frequently associated with various autosomal dominant mutations in multiple genes encoding sarcomeric proteins. These include mutations in genes encoding cardiac troponins -I (*TNNI3*), -T (*TNNT2*), and -C (*TNNC1*), α-tropomyosin (*TPM1*), myosin light chains *(MYL2* and *MYL3*), and *MYH6* (myosin heavy chain 6, also referred to as α-myosin heavy chain) [7]. Notably, mutations in genes encoding cardiac β-myosin heavy chain (*MYH7*) and cardiac myosin binding protein-C (*MYBPC3*) have more commonly been linked to HCM [8], collectively accounting for approximately 40–50% of familial cases [9,10,11]. These mutations give rise to a wide spectrum of phenotypes, ranging from normal heart or mild hypertrophy to severe hypertrophy, causing an increased susceptibility to life-threatening ventricular arrhythmias [12,13]. Beyond these classical sarcomeric mutations, *MYH7* variants are also implicated in 40% of congenital HCM cases, and both *MYH7* and *MYBPC3* have been linked to other cardiomyopathies such as dilated cardiomyopathy (DCM) and left ventricular non-compaction (LVNC). Recent insights have revealed that more than 1000 mutations across around 100 genes, spanning diverse subcellular systems, lead to the pathogenesis of cardiomyopathies. This genetic heterogeneity highlights the complexity of the cardiac remodeling process and underscores a shift in diagnostic strategies, from a morphology-based to a genotype-guided approach. The advent of high-throughput sequencing technologies has facilitated earlier detection of pathogenic variants, often before the onset of clinical symptoms, enhancing both risk stratification and family screening. However, the growth of genetic data requires specialized genetic counseling and meticulous interpretation to guide personalized patient care and familial risk assessment [14].

The myosin binding protein-C (MyBP-C) family comprises a group of sarcomeric proteins recognized for their role as modulators of myofilament contractility. There are three isoforms of MyBP-C, each produced by a different gene and exhibiting unique expression patterns in striated muscle. The *MYBPC3* gene is located on chromosome 11 and encodes cardiac MyBP-C (cMyBP-C), a protein with a globular domain structure similar to the immunoglobulin (Ig) or fibronectin-III (Fn3) protein families. cMyBP-C comprises eleven domains (C0–C10), including a myosin-binding motif (M-domain) located between C1 and C2 domains. In addition to a Pro-Ala-rich linker connecting the C0 and C1 domains, the M-domain plays an important role in facilitating the interaction between C1 and C2 [15]. The C0 domain has a typical structure representative of the IgI fold, found in most MyBP-C proteins, formed by a β-sandwich consisting of two β-sheets [16]. This domain is part of the thick filament in striated muscles, contributing to sarcomere formation and the regulation of muscle contraction [15]. cMyBP-C has a dual effect on sarcomere contraction, which is contingent upon the local calcium concentration [Ca^2+^] regulating its N-terminus interaction with actin and myosin [3]. Specific *MYBPC3* mutations have been linked to a delayed onset of HCM, displaying lower penetrance, reduced hypertrophy, and a more favorable prognosis relative to other severe gene mutations [1]. The growing body of evidence linking cMyBP-C to HCM highlights the need to deepen our understanding of the molecular mechanisms by which mutations in the *MYBPC3* gene drive disease pathogenesis [10].

This study focuses on the biochemical and biophysical characterization of the C0–C2 region of *MYBPC3* harboring two HCM-associated missense mutations (c.706A>G and c.1000G>A)*,* resulting in amino acid substitutions (p.S236G and p.E334K), respectively. The substituted amino acids are located in the C1 and M-domains of cMyBP-C protein, respectively. The p.E334K mutation was initially detected in Japanese patients suffering from HCM [17], while the p.S236G was found in HCM patients from China [18] and Finland [19]. Nevertheless, the pathogenicity of these variants remains inconclusive, primarily due to the absence of a clear phenotype-genotype relationship. Although the p.E334K mutation is highly prevalent among patients, some p.E334K carriers have displayed cardiac dilation and dysfunction [17]. In the case of p.S236G, prior investigations have shown that this mutation does not segregate with the HCM phenotype. p.S236G carriers appear to be clinically unaffected. However, this mutation was detected in families with other cMyBP-C defects. Consequently, p.S236G has been generally classified as a neutral polymorphism [19]. This classification is further supported by the Clinvar archive (https://www.ncbi.nlm.nih.gov/clinvar/), (accessed on 10 March 2025), which categorizes the p.S236G mutation as benign. Additionally, it has been suggested that this mutation could induce a mild, non-pathological hypertrophy that could provide certain advantages to elite athletes [20]. Herein, we assess the impact of these two *MYBPC3* mutations at the protein level, on the tertiary structure and functional properties, by expressing the corresponding C0–C2 domain of these *MYBPC3* mutants as recombinant proteins. Subsequently, we employ circular dichroism (CD), differential scanning calorimetry (DSC), fluorescence spectroscopy, and actin-binding assays to directly compare the molecular properties of these cMyBP-C mutants (cMyBP-C^S236G^ and cMyBP-C^E334K^) to those of the wild-type cMyBP-C (cMyBP-C^WT^) protein.

## 2. Materials and Methods

### 2.1. Plasmid Construction

For the generation of the pETMM11-cMyBP-C^WT^, cMyBP-C^S236G^, and cMyBP-C^E334K^ C0–C2 constructs, cDNAs corresponding to human *MYBPC3* wild-type (NM_000256.3) and the two *MYBPC3* mutants were used as templates to amplify the regions corresponding to C0–C2 domains of cMyBP-C by polymerase chain reaction (PCR). Phusion polymerase (Thermo Fisher Scientific, Waltham, MA, USA) and the appropriate primers to incorporate 5′-EcoRI and 3′-SalI sites were used for the PCR. The amplified constructs were cloned into the pETMM11 expression vector. The primers used for the amplification of the cMyBP-C^WT^, cMyBP-C^S236G^, and cMyBP-C^E334K^ constructs were 5′-TCGGGAATTCGATGCCTGAGCCGGGGAAGAAGCC-3′ (forward) and 5′-GAGGGTCGACTCACTCTTTCACAAAGAGCTCCGTGC-3′ (reverse).

### 2.2. In Silico Pathogenicity Prediction Tools

Three computational tools based on in silico prediction algorithms were employed to investigate the structural and functional changes in cMyBP-C^S236G^ and cMyBP-C^E334K^, as well as to evaluate the potential deleterious effects on their interactions with other proteins [21,22].

The three tools used for predicting the pathogenicity of the mutations include Sorting Intolerant From Tolerant (SIFT), Polymorphism Phenotyping v2 (Polyphen-2), and DDmut:

- SIFT: Performs multiple sequence alignment of the query protein sequence to generate a SIFT score. Scores range from 0 to 1, with a score higher than 0.05 generally indicating a “tolerated” mutation, which likely has a mild effect on protein function and is not considered pathogenic. Conversely, a score lower than 0.05 suggests a pathogenic mutation. Specifically, when the score is closer to 1, the mutation is benign, while a score between 0.05 and 0.1 may indicate a potentially damaging but not highly severe mutation. A score closer to 0 corresponds to a pathogenic mutation causing a severe impact on protein function [23];
- Polyphen-2: Utilizes protein sequences to extract structural annotations from transcriptome databases, classifying pathogenicity into benign, possibly damaging, or probably damaging categories [24]. Benign demonstrates that the mutation is unlikely and has no potential to be harmful or pathogenic;
- DDmut: Predicts the impact of single or multiple mutations on protein stability and determines the relative b-factor [19]. This tool presents structural information regarding the mutation, based on the predicted change in free energy (ΔΔG) and b-factor.

All the aforementioned tools collectively aid in assessing the potential pathogenicity of mutations, offering valuable insights for medical research and clinical applications [25].

### 2.3. Protein Expression and Purification

Following confirmation of successful cloning, the generated pETMM11-cMyBP-C^WT^, cMyBP-C^S236G^, and cMyBP-C^E334K^ C0–C2 constructs were transformed into competent *E. coli* [BL21- CodonPlus (DE3)-RILP; Stratagene] cells. The bacterial cells were cultured at 37 °C until the optical density at 600 nm reached 0.6, then induced with 0.1 mM isopropyl-β-d-thiogalactopyranoside (IPTG) for 18 h at 16 °C. Bacterial cell pellets were harvested by centrifugation at 6000× *g* for 15 min at 4 °C. Recombinant cMyBP-C^WT^, cMyBP-C^S236G^, and cMyBP-C^E334K^ proteins were then purified by immobilized metal affinity chromatography using a Ni^2+^-chelating resin (QIAGEN), as previously described [26]. The purity of the eluted recombinant proteins was analyzed using SDS-PAGE, followed by Coomassie brilliant blue staining.

### 2.4. Circular Dichroism

The circular dichroism spectra in the far-UV region for cMyBP-C^WT^, cMyBP-C^S236G^, and cMyBP-C^E334K^ recombinant proteins were acquired using a JASCO-1100 spectropolarimeter (JASCO Corporation, Hachioji, Tokyo, Japan), which was equipped with a Peltier-type cell holder for precise temperature regulation. These spectra were measured between 195 and 260 nm at two distinct temperatures (25 °C and 85 °C) utilizing a SUPRASIL quartz cell from Hellma, Germany, with a path length of 1 mm. The experimental setup involved loading 250 μL aliquots of 0.2 mg/mL of protein solution in 1x PBS buffer, pH 8.0, into the cuvette. The following parameters were employed for the far-UV measurements: 0.1 nm step size, 50 nm/min scan speed, 1 nm bandwidth, and 8 accumulations. After each measurement, pure buffer spectra were obtained under identical conditions, subtracted from the protein scans, and the resulting data in mdeg units were normalized to molar ellipticity (deg·cm^2^/dmol units). The estimation of secondary structure percentages was carried out by analyzing the normalized spectra using CDNN software v2.1, (2015) link: https://cdnn.software.informer.com/2.1/, accessed on 21 April 2025 [27,28].

### 2.5. Differential Scanning Calorimetry

Thermal unfolding analyses of cMyBP-C^WT^, cMyBP-C^S236G^, and cMyBP-C^E334K^ were conducted using a Nano DSC differential scanning calorimeter (TA Instruments, New Castle, DE, USA). The protein samples were prepared in 1× PBS buffer, pH 8.0, at a final concentration of 0.8 mg/mL. Prior to each protein measurement, multiple scans were performed with both DSC cells filled with buffer, ensuring optimal baseline repeatability. Protein and buffer solutions were thoroughly degassed before loading into the device to avoid bubble formation. Measurements of excess heat capacity at constant pressure as a function of temperature (T) were collected in the range of 35–70 °C using a scan rate of 1.5 °C/min. After completing the first run and cooling the sample back to 35 °C, a second run was performed with identical settings to assess the reversibility of the transition. In all cases, the thermal unfolding of the proteins exhibited poor reversibility, with less than 50% of the protein population returning to the native state as determined by direct comparison of the area under the two sequential DSC traces. Finally, the corresponding buffer scans were subtracted from the protein thermograms, and the final data were normalized according to protein concentration to obtain the excess molar heat capacity function <C_p_>. DSC data analysis was performed using the instrument’s native NanoAnalyze software: v4.0.2.0 (2024), TA Instruments, DE, USA. The characteristic temperature (T_m_) and enthalpy (ΔH_unf_) of the thermal unfolding process were determined from the maximum of the peak and the integrated area under the peak, respectively, after subtraction of the sigmoidal chemical baseline of the transition.

### 2.6. Chemical Denaturation Experiments

Chemical denaturation profiles of cMyBP-C^WT^, cMyBP-C^S236G^, and cMyBP-C^E334K^ were obtained through the incremental addition of small aliquots of a concentrated chaotrope, while monitoring changes in fluorescence emission within the 300–450 nm spectrum after excitation at 295 nm. Specifically, 0.1 mg/mL of protein in 1× PBS buffer at pH 8.0 was placed in a 3.5 mL quartz cuvette (Macro Cell 101-QS, Helma GmbH & Co. KG, Müllheim, Germany), with gradual additions of an 8 M guanidinium chloride (GuHCl) solution. The protein concentration was maintained by supplementing the appropriate volume of a concentrated protein solution to the cuvette. Subsequently, the mixture was stirred and left to equilibrate at room temperature until a new steady state was reached (approximately 15 min). Fluorescence measurements in the near-UV/visible range were performed utilizing a Horiba Fluoromax 4 Spectrofluorometer (HORIBA Advanced Techno—Kyoto, Japan), equipped with a xenon short-arc lamp (Ushio). The titration process continued until a final concentration of 4.5 M GuHCl was achieved. Mock titrations were conducted to remove contributions from GuHCl or the buffer from the final protein spectrum. Finally, the experimental data are presented as weighted average wavelength change Δλ_WA_ versus GuHCl concentration [D] and fitted to a two-state unfolding model of the following form:

$${\Delta \lambda }_{WA}=\frac{{(a}_{N}+b_{N}\left[D\right])+{(a}_{D}+b_{D}\left[D\right])\cdot exp\frac{\left(m_{DN}\cdot \left(\left[D\right]-{\left[D\right]}_{50\%}\right)\right)}{RT}}{1+exp\frac{\left(m_{DN}\cdot \left(\left[D\right]-{\left[D\right]}_{50\%}\right)\right)}{RT}}$$

where α_N_ is the signal of the native state at 0 M denaturant, b_N_ is the signal slope (dα_N_/d[D]) at the native state, α_D_ and b_D_ are the corresponding quantities for the denatured state, m_DN_ is a proportionality constant (−∂(ΔG_DN_)/∂[D]), and [D]_50%_ is the denaturant concentration at which the protein is 50% unfolded. The nonlinear least-squares fit of this equation to the experimental data allows the estimation of the [D]_50%_ and mDN parameters as along with their standard deviations. The free energy change between the native and the denatured state (ΔG_DN_) can then be calculated from the following equation:

ΔG_DN_ = m_DN_ · [D]_50%_

### 2.7. Molecular Dynamics Simulations

Molecular dynamics (MD) simulations were conducted by using GROMACS version 2019.2 [29,30] and the CHARMM36 force field [31,32] on the cMyBP-C^WT^, cMyBP-C^S236G^, and cMyBP-C^E334K^ protein fragments. Protein Data Bank (https://www.rcsb.org/, accessed on 10 March 2025) entries 3CX2 [33] and 5K6P [34], corresponding to cMyBP-C residues 151-258aa and 315-451aa respectively, were used as wild-type structures for the MD simulations. Mutations were introduced to each wild-type structure using the Protein Builder tool of the Molecular Operating Environment software version 2022.02 (Molecular Operating Environment (MOE) (2014), Montreal, QC, Canada). Initially, each protein fragment was solvated in a triclinic unit cell with simple point charge (SPC) [35] water molecules at a 10 Å marginal radius. Ten water molecules were replaced by sodium counter ions to nullify the total charge of each system. The ionization states of the residues were calculated at pH 7.0, assuming that all histidine residues remain in a neutral deprotonated state. The solvated protein systems then underwent energy minimization for 5000 steps employing the steepest descent algorithm. Following the energy minimization, all systems were stabilized utilizing a canonical ensemble (NVT) at 300 K, followed by the isobaric-isothermal ensemble (NPT) at 300 K and 1 bar pressure for 500 ps, respectively. The system pressure and temperature were held constant at 1 bar and 300 K, respectively, with a coupling time constant of 1.0 ps. A 1.0 nm cut-off was applied to all van der Waals and Coulomb interactions within the simulation boundaries. Subsequently, all protein systems underwent a 50 ns molecular dynamics simulation. Trajectory data were gathered at intervals of 500 ps. Utilizing the gmx cluster analysis tool in GROMACS and the Gromos method [36], the MD simulation trajectories were clustered to identify the most representative structures of the ensemble. The root-mean-square deviation (RMSD) and root-mean-square fluctuation (RMSF) were calculated from the trajectory files using the gmx rms and gmx rmsf tools in GROMACS. The radius of gyration (k) and the solvent-accessible surface area (SASA) were computed for each simulation step using the gmx gyrate and gmx sasa commands, respectively.

### 2.8. Co-Sedimentation Cardiac Actin-Binding Assays

Co-sedimentation assays were performed as previously described [37] using the Actin-binding Protein Biochem Kit (Cytoskeleton, Denver, CO, USA, Cat # BK001) and bovine cardiac actin (Cytoskeleton, Cat # AD99-B). Bovine cardiac actin (c-actin) was prepared in General Actin Buffer (5 mM Tris-HCl pH 8.0, 0.2 mM CaCl_2_) (Cytoskeleton, Cat # BSA01) and supplemented with 0.2 mM ATP and 0.5 mM DTT prior to use. Briefly, 30 µM of c-actin was incubated with 2 µM of each cMyBP-C^WT^ recombinant protein for 30 min at room temperature. After incubation, samples were centrifuged at 14,000× *g* for 1 h at 24 °C. Then the supernatants were carefully removed, mixed with 10 mL of 5× Laemmli reducing-sample buffer, and analyzed by SDS-PAGE electrophoresis followed by Coomassie brilliant blue staining. The relative binding of c-actin to the various recombinant proteins was then estimated by the intensities of the bands appearing on the stained gels, which were measured by densitometric analysis. Finally, the data were analyzed using GraphPad Prism 7.

## 3. Results

### 3.1. In Silico Analysis of Pathogenicity and Structural Consequences of S236G and E334K Mutations in cMyBP-C

To gain an initial insight into the potential impact of p.S236G and p.E334K mutations, three different bioinformatics tools were used (SIFT, Polyphen2, and DDmut) [23,24,25] to predict structural changes and potential pathogenicity, using the protein’s primary structure (Uniprot database entry: Q14896). Table 1 summarizes the predictions of these algorithms for the p.S236G and p.E334K amino acid substitutions on cMyBP-C function, pathogenicity, as well as structural stability. The PolyPhen-2 analysis of MyBPC3 mutations provides three key values, a low Score (Near 0) indicating that the p.S236G mutation is predicted to be benign, while a high score (0.874) for the p.E334K suggesting that this substitution is likely to be harmful and could negatively affect the protein’s structure and/or function. Higher sensitivity reflects the reliability of Polyphen2 in identifying deleterious mutations from benign ones. However, the lower specificity, as predicted for the p.S236G substitution (0.15 or 15%), means that only 15% of truly benign mutations are correctly predicted as benign, while 85% might be misclassified as potentially harmful. Conversely, a higher specificity, as predicted for the p.E334K substitution, reflects the software’s reliability in minimizing false positives. Nonetheless, there is speculation that this substitution could induce protein structure destabilization, as suggested by the predicted free energy change difference (ΔΔG) of −0.39 kcal/mol. The p.E334K substitution is classified as tolerated with a potential damaging effect according to Polyphen2, while DDmut predicts no significant destabilizing effect on cMyBP-C structure (ΔΔG = 0.02 kcal/mol). The b-factor value predictions by DDmut can also be used to evaluate the impact of the mutations on protein dynamics; lower b-factor values indicate relatively fixed positions of the atoms, suggesting a more rigid protein structure, whereas higher b-factor values indicate greater atom mobility and flexibility. In this case, p.E334K has a lower b-factor value compared to p.S236G (−0.33 Å2 and −0.12 Å2, respectively), implying different dynamic behavior of these two variants.

**Figure 1:**
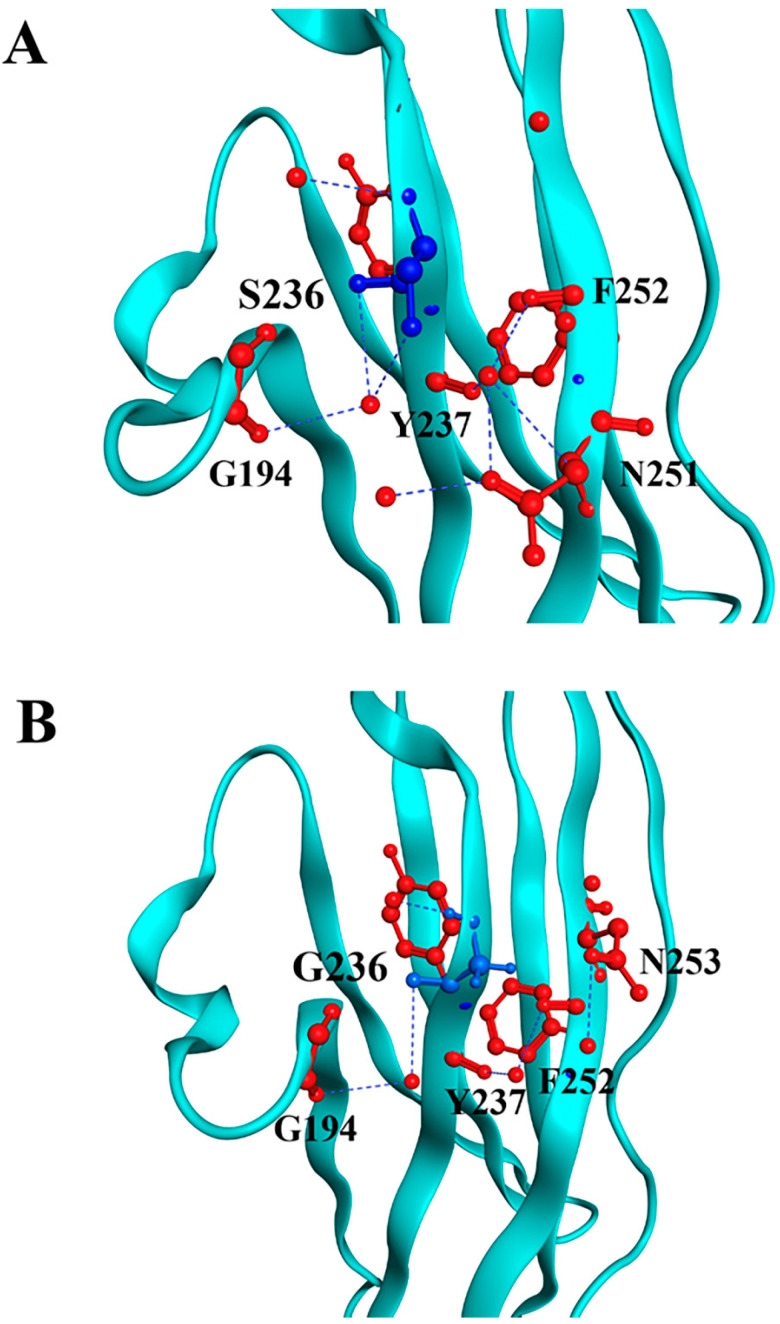
Molecular modeling of cMyBP-C^WT^ and cMyBP-C^S236G^ local interactions. Structural illustrations of the interactions of the wild-type S236 (panel **A**) and the mutant G236 (panel **B**) residues with surrounding amino acids within the cMyBP-C structure. The targeted residue, in position 236, is highlighted in blue, while surrounding residues are depicted in red. Interactions are indicated as blue lines. In silico modeling was conducted using the crystal structure 3CX2 [32] from the Protein Data Bank depository (PDB, https://www.rcsb.org/, accessed on 10 March 2025) as a template.

Through in silico modeling, we can visualize both substitutions and their interactions with the neighboring residues. For the p.S236G substitution, the model revealed that the surrounding residues near position 236 remain similar. However, substituting serine with glycine weakens its interaction with G194 (Figure 1).

**Figure 2:**
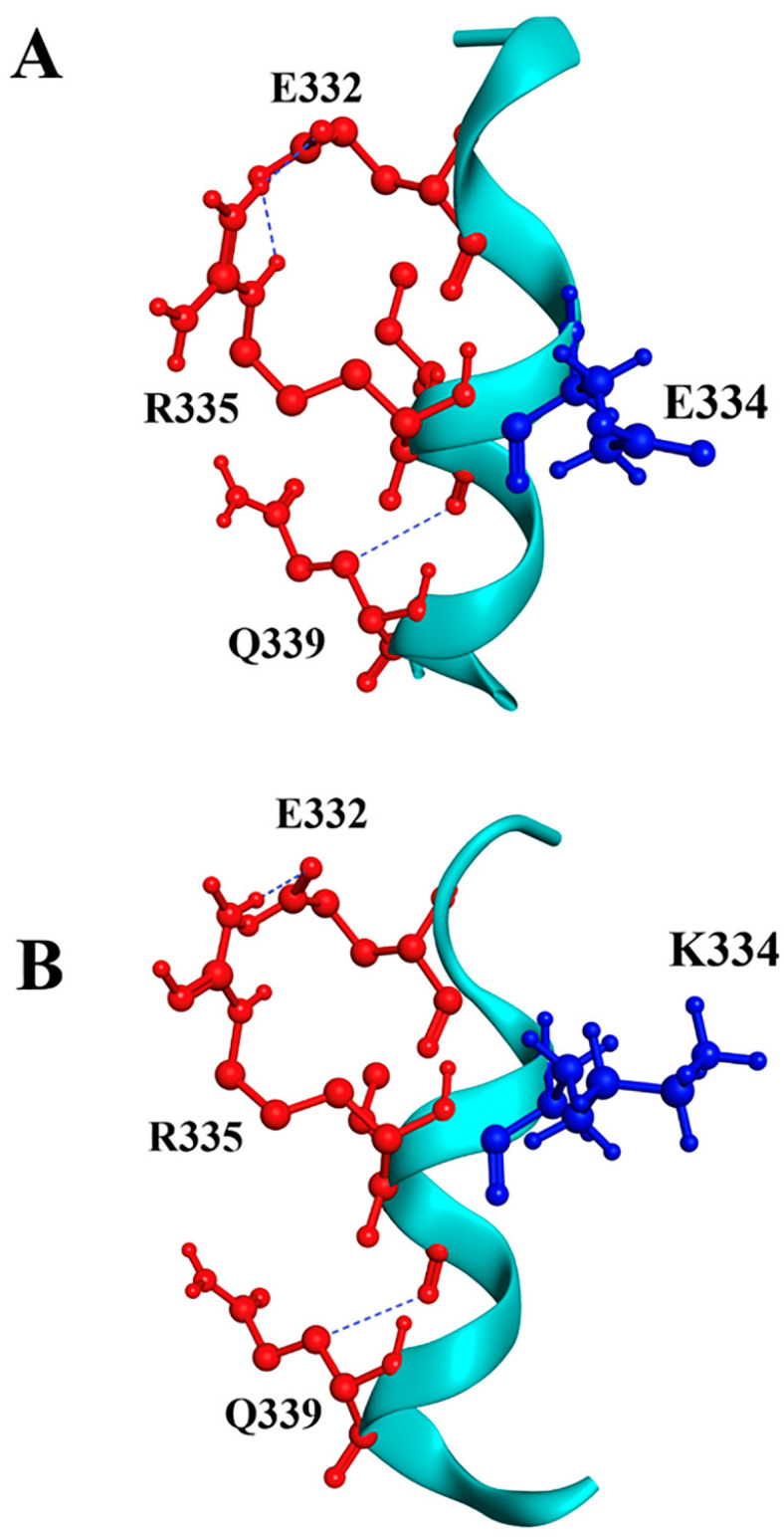
Molecular modeling of cMyBP-C^WT^ and cMyBP-C^E334K^ local interactions. Structural illustrations of the interactions of the wild-type E334 (panel **A**) and the mutant K334 (panel **B**) residues with surrounding amino acids within the cMyBP-C structure. The targeted residue, in position 334, is highlighted in blue, while surrounding residues are depicted in red. Interactions are indicated as blue lines. In silico modeling was conducted using the crystal structure of 5K6P [33] from the Protein Data Bank depository (PDB, https://www.rcsb.org/, accessed on 10 March 2025) as a template.

For the p.E334K substitution, our prediction shows similar surrounding residues near position 334. In the wild-type protein, several salt bridges are observed, notably E332-R335 and E334-R346, using VMD software (version 1.9.4a53, 29 June 2021). Substituting E334 with lysine may disrupt the salt bridge with R346, which could reduce protein stability and increase susceptibility to unfolding and fast denaturation (Figure 2). This analysis strongly suggests a potential negative effect of the p.E334K mutation on the structure-function interplay of cMyBP-C.

### 3.2. Generation of Recombinant Wild-Type and Mutant cMyBP-C Proteins

**Figure 3:**
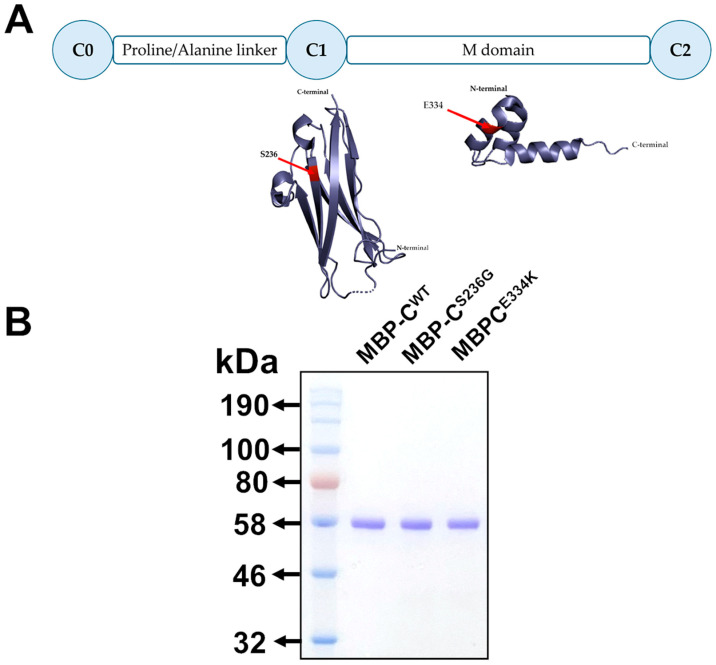
Expression of isolated C0–C2 domains of cMyBP-C^WT^, cMyBP-C^S236G^, and cMyBP-C^E334K^ as recombinant proteins. (**A**). Schematic representation of the C0–C2 domains of cMyBP-C expressed as recombinant proteins. The corresponding secondary structures are shown with the substituted residues, S236G and E334K, highlighted in red. (**B**). Analysis of affinity-chromatography purified recombinant C0–C2 domain fragments corresponding to the cMyBP-C^WT^, cMyBP-C^S236G^, and cMyBP-C^E334K^ variants by 12% SDS-PAGE followed by Coomassie Brilliant Blue staining.

To explore the potential structural and functional effects of the p.S236G and p.E334K amino acid substitutions within cMyBP-C at the protein level, cMyBP-C^WT^, cMyBP-C^S236G^, and cMyBP-C^E334K^ isolated C0–C2 domains (Figure 3A) were cloned into the pETMM11 plasmid vector, expressed as recombinant proteins utilizing a prokaryotic expression system and purified by affinity chromatography, as described in the Materials and Methods section. SDS-PAGE analysis of the resultant recombinant proteins revealed a single band with mobility consistent with the predicted molecular weight (~54 kDa) for all expressed proteins, confirming their homogeneity (Figure 3B).

### 3.3. Circular Dichroism (CD) Spectroscopy and Thermal Stability Analysis of cMyBP-C Variants

**Figure 4:**
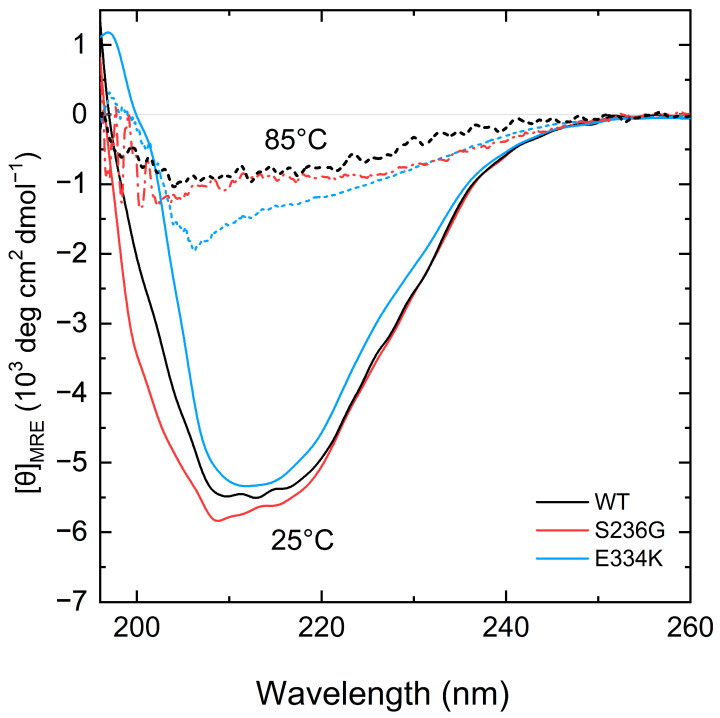
CD spectra measurements of the recombinant C0–C2 cMyBP-C fragments. Far-UV CD spectra of recombinant proteins corresponding to 0.2 mg/mL of cMyBP-C^WT^ (black), cMyBP-C^S236G^ (red), and cMyBP-C^E334K^ (blue) at 25 °C (solid lines). After heating the same samples at 85 °C, all proteins adopt a similar unfolded structure (dashed lines).

To elucidate any potential structural impact of the p.S236G and p.E334K substitutions, circular dichroism was employed to analyze the far-UV CD spectra (Figure 4) and determine the secondary structural content of all recombinant protein fragments at 25 °C (Table 2).

Analysis of the CD spectra showed notable changes at the secondary structure level for both cMyBP-C^S236G^ and cMyBP-C^E334K^ recombinant proteins in comparison with cMyBP-C^WT^ (Figure 4). Furthermore, by heating the protein samples at 85 °C, all proteins adopted a random coil conformation without significant remaining secondary structure motifs (Figure 4).

**Figure 5:**
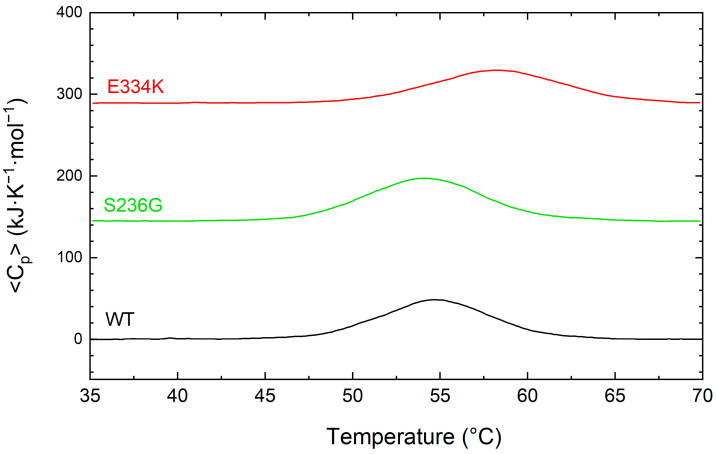
DCS profiles for the thermally induced denaturation of recombinant C0–C2 cMyBP-C fragments. All profiles were obtained at identical solution conditions, and the concentration of all recombinant proteins [cMyBP-C^WT^ (black), cMyBP-C^S236G^ (green), and cMyBP-C^E334K^ (red)] was 0.8 mg/mL. The DSC traces shown in this figure correspond to the first heating run from 35 °C to 70 °C and have been vertically translated for reasons of clarity.

For a more comprehensive examination of the thermal unfolding behavior of these domains, the cMyBP-C^WT^ (black), cMyBP-C^S236G^ (red), and cMyBP-C^E334K^ protein samples were further analyzed using differential scanning calorimetry (DSC), and the results are shown in Figure 5 and summarized in Table 3. Our data indicates a two-step unfolding mechanism for the protein fragments, characterized by an enthalpy change in the region of 375 to 432 kJ/mol and a Tm ranging from 58.4 °C to 54.2 °C (Table 3). Compared to cMyBP-C^WT^, cMyBP-C^S236G^ and cMyBP-C^E334K^ C0–C2 protein fragments showed a ~6 and ~13% reduced enthalpy, respectively, consistent with small structural modifications within the molecular arrangement. This is in excellent agreement with our CD measurements for these two variants. It is also worth noting that the thermal unfolding profile of cMyBP-C^E334K^ exhibited an increased thermal stability for this mutant in comparison to cMyBP-C^WT^ (Tm = 58.4 vs. 54.7 °C, respectively), (Figure 5 and Table 3).

### 3.4. Chemical Denaturation Profiles for cMyBP-C C0–C2 Proteins

**Figure 6:**
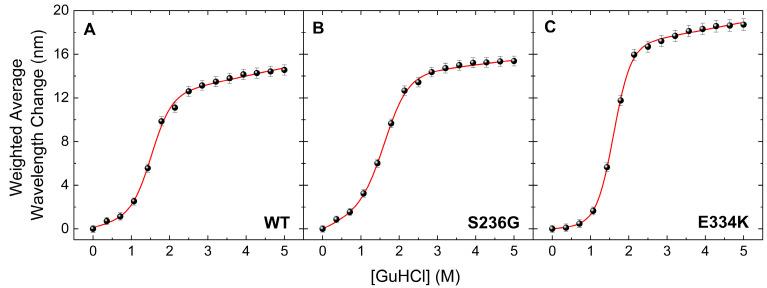
Chemical denaturation profiles of the C0–C2 fragments of cMyBP-C determined by steady-state fluorescence spectroscopy. Data are plotted as the weighted average wavelength of the protein fluorescence emission spectra at different GuHCl concentrations, after excitation at 295 nm. All cMyBP-C protein fragments showed a completely reversible transition, described by a sigmoidal two-state denaturation curve for the fraction of unfolding. (**A**) Denaturation profile of cMyBP-C^WT^ (**B**) Denaturation profile of cMyBP-C^S236G^. (**C**) Denaturation profile of cMyBP-C ^E334K^.

The thermal transition reversibility for all cMyBP-C^WT^, cMyBP-C^S236G^, and cMyBP-C^E334K^ proteins was found to be less than 60%, as determined by comparing the ΔHunf values from the first and second DSC run of the same sample. This suggests that deriving meaningful thermodynamic stability data from the obtained thermograms is not feasible, as there is a significant kinetic component involved. To further investigate the thermodynamic stability of these two cMyBP-C variants (cMyBP-C^S236G^ and cMyBP-C^E334K^), we performed chemical denaturation experiments by measuring fluorescence emissions of the proteins under different concentrations of the chemical denaturant GuHCl in the solution. Results indicated a notable decrease in the free energy change (ΔG_DN_) for cMyBP-C^S236G^, whereas intriguingly, cMyBP-C^E334K^ exhibited an increased ΔG_DN_ compared to cMyBP-C^WT^ (Figure 6 and Table 4). Considering that ΔGDN represents the energy barrier separating the fully folded and fully unfolded states of the protein, this can be directly interpreted as an increase in the thermodynamic stability of cMyBP-C^E334K^. Interestingly, the thermal unfolding of cMyBP-C^E334K^ monitored by DSC also showed increased thermal stability for this mutant compared to cMyBP-C^WT^ (Tm = 58 °C vs. 54 °C, respectively). Thermal and chemical unfolding processes might follow a different pathway on the energy landscape of the protein, but in both cases, p.E334K seems to increase the stability of the protein fragment. Our findings suggest that the p.E334K substitution may result in a more rigid structure compared to the wild-type isoform, potentially affecting the dynamics and flexibility of the molecule, which in turn may impair interaction with other important protein binding targets.

### 3.5. Analysis of MD Trajectories

**Figure 7:**
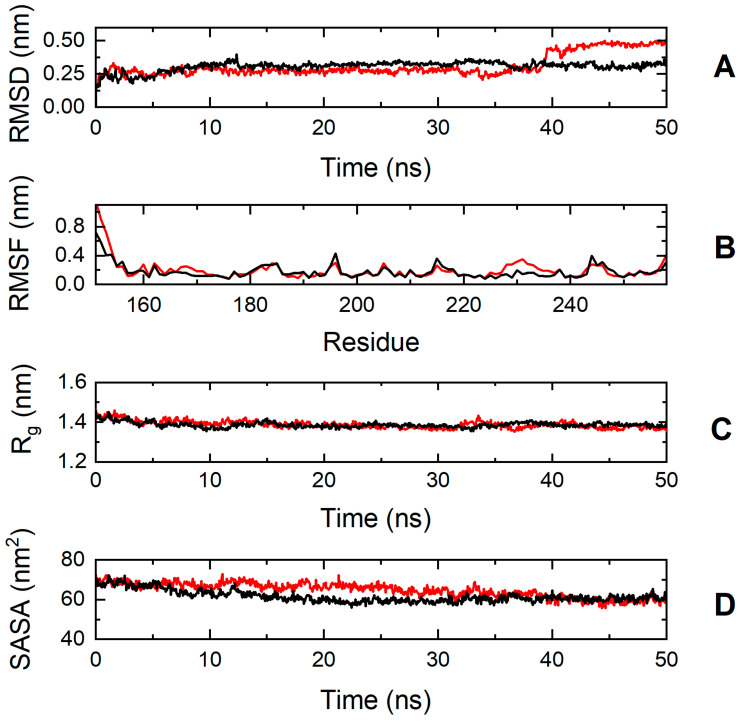
Trajectory analysis of cMyBP-C^WT^ (black, ―) and cMyBP-C^S236G^ (red, ―) protein fragments (residues 151-258). (**A**) Root mean square deviation (RMSD) of the protein backbone atoms as a function of MD simulation time. (**B**) Root-mean-square-fluctuations (RMSFs) of individual residues for the duration of the simulation. (**C**) Total radius of gyration (R_g_) of the protein backbone atoms as a function of MD simulation time. (**D**) Solvent accessible surface area (SASA) of the protein as a function of MD simulation time.

To explore further the impact of the p.S236G and p.E334K mutations on protein dynamics, a series of molecular dynamics simulations were conducted for cMyBP-C^WT^, cMyBP-C^S236G^, and cMyBP-C^E334K^ proteins. Analysis of the simulation trajectories for cMyBP-C^WT^ and the cMyBP-C^S236G^ mutation uncovered a similar behavior for both proteins in terms of root-mean-square deviation (RMSD), root-mean-square-fluctuations (RMSFs), radius of gyration (Rg), and solvent-accessible surface area (SASA) throughout the simulation duration, indicating a minimal influence of the mutation on the protein dynamics and overall folding (Figure 7).

**Figure 8:**
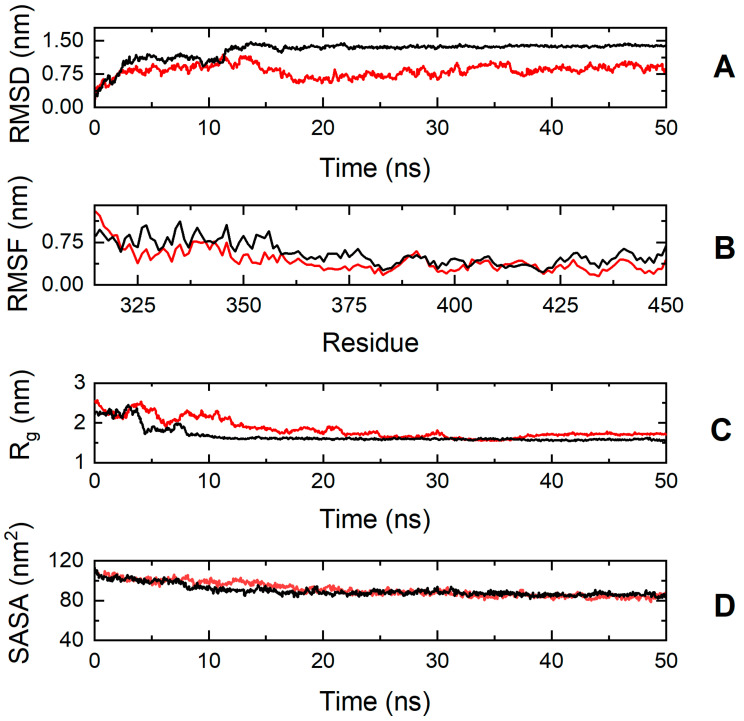
Trajectory analysis of cMyBP-C^WT^ (black, ―) and cMyBP-C^E334K^ (red, ―) protein fragments (residues 315-451). (**A**) Root mean square deviation (RMSD) of the protein backbone atoms as a function of MD simulation time. (**B**) Root-mean-square-fluctuations (RMSFs) of individual residues for the duration of the simulation. (**C**) Total radius of gyration (R_g_) of the protein backbone atoms as a function of MD simulation time. (**D**) Solvent accessible surface area (SASA) of the protein as a function of MD simulation time.

On the contrary, the simulation results suggest that the cMyBP-C E334K mutation has a more significant impact on protein structure and dynamics (Figure 8). The cMyBP-C^E334K^ segment shows a very different RMSD profile compared to the native protein, with a backbone deviation change (RMSD^WT^—RMSD^E334K^) ranging from −0.2 to 0.84 nm during the simulation.

The effect of the mutation on protein dynamics is also apparent when analyzing the RMSF parameter of the trajectories, which denotes the average deviation of atomic placements from their average positions throughout the simulation. From the RMSF diagram (Figure 8, Panel B), it can be deduced that residue-level fluctuations for the mutant structure were notably reduced in comparison to the native structure, particularly for residues found within positions 320–340, 345–380, and 425–451. This loss of flexibility for the cMyBP-C^E334K^ protein is in excellent agreement with the chemical denaturation experiments, indicating that cMyBP-C^E334K^ is thermodynamically more stable, but also more rigid when compared to cMyBP-C^WT^.

### 3.6. Impact of p.S236G and p.E334K Mutations on the Interaction of cMyBP-C with Cardiac Actin

**Figure 9:**
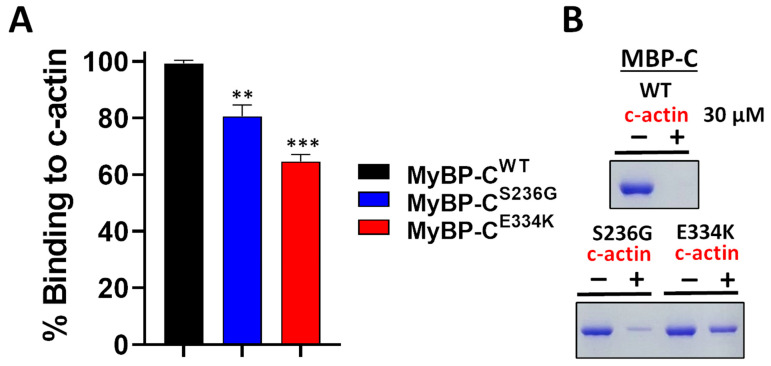
Impact of S236G and E334K mutations on the actin-binding properties of cMyBP-C. Co-sedimentation binding assays of recombinant cMyBP-C^WT^, cMyBP-C^S236G^, and cMyBP-C^E334K^ C0–C2 protein fragments, and c-actin. Recombinant cMyBP-C proteins (2 µM) were incubated with c-actin at RT for 30 min and centrifuged at 14,000× *g* for 1 h. The amount of recombinant cMyBP-C proteins in the supernatant (unbound fraction) was analyzed by SDS-PAGE and quantified by Coomassie brilliant blue staining and densitometric analysis. (**A**) Densitometric analysis and percent normalization of the binding of recombinant cMyBP-C proteins to 30 μM of c-actin. (**B**) Representative SDS-PAGE gels and Coomassie brilliant blue staining following cMyBP-C recombinant protein-actin co-sedimentation assays showing the amounts of cMyBP-C recombinant proteins in supernatant fractions (unbound fraction) before (−) and after (+) co-sedimentation with 30 µM of c-actin. Statistically significant differences (asterisks) were analyzed by comparison to the binding of cMyBP-C^WT^ using an unpaired Student’s *t*-test (*n* = 3, ** *p* < 0.01 and *** *p* < 0.01, GraphPad Prism 7 software was used to analyze the data).

Several studies have reported that recombinant cMyBP-C C0–C2 domains interact with actin within the sarcomere [26,27,28]. This critical interaction plays a pivotal role in cardiac muscle contraction. To assess the impact of the S236G and E334K substitutions on the cMyBP-C actin-binding properties, we employed a co-sedimentation assay that we have previously optimized and described elsewhere [26]. In this assay, c-actin co-sediments due to its fibrous nature, while non-fibrous or non-interacting proteins remain in the supernatant fraction. Following previous optimization experiments, we showed that 2 µM of cMyBP-C^WT^ can bind to 30 µM actin to saturation (Figure 9). The co-sedimentation assays were repeated with cMyBP-C^S236G^ and cMyBP-C^E334K^, and the supernatants (unbound fractions) were analyzed by SDS-PAGE gel electrophoresis, followed by Coomassie brilliant blue staining and densitometric analysis. Our findings reveal that although cMyBP-C^S236G^ had a moderately (~19%) reduced relative actin-binding affinity, the cMyBP-C^E334K^ mutant showed a more significantly (~35%) reduced relative binding affinity to actin (Figure 9B). This large reduction in binding affinity could potentially be attributed to an indirect consequence of the structural modifications induced by the E334K amino acid substitution.

## 4. Discussion

The aim of this study was to investigate the structural and functional effects of two HCM-associated genetic mutations, p.S236G and p.E334K, located within the N-terminal C0–C2 domains of the cMyBP-C protein. Below, we discuss our findings in the context of structural changes, interaction with actin, and their implications for HCM pathogenesis.

In silico modelling indicated that these mutations, especially the p.E334K mutation, have the potential to modify the secondary and tertiary structure of the C0–C2 domains, consequently affecting the overall folding and probably the stability of the cMyBP-C protein. To address the validity of these computational calculations, a series of biophysical and biochemical experiments were carried out. Structural changes were monitored by CD spectroscopy, while functional effects were monitored by co-sedimentation assays to determine the actin binding affinity of cMyBP-C^S236G^ and cMyBP-C^E334K^ compared to cMyBP-CWT.

CD spectroscopy and DSC analysis demonstrated that both substitutions affect the secondary structure of the C0–C2 domains. Notably, the p.E334K substitution increased thermal stability, likely due to enhanced rigidity and impaired folding dynamics, while p.S236G exhibited decreased stability compared to the wild-type protein. Remarkably, cMyBP-C^S236G^ exhibited decreased stability compared to the wild-type, as indicated by ΔGDN of the denaturation process, while cMyBP-C^E334K^ unfolded at higher chaotropic concentrations than either cMyBP-C^WT^ or cMyBP-C^S236G^. The resulting structural modifications between these variants are attributed to their distinct locations within the cMyBP-C protein. The S236G resides in a less conserved region, resulting in milder effects, whereas E334K belongs to a highly conserved tri-helix bundle that is critical for structural integrity and functional interactions with other biological targets.

Our co-sedimentation assays demonstrated that both mutations significantly reduced actin-binding affinity, with cMyBP-C^E334K^ displaying the most pronounced effect.

These results are in excellent agreement with other studies, such as showing diminished actin binding by the double mutant cMyBP-C^E258K-E441K^ [37]. Similarly, research on the mouse cMyBP-C^E330K^ C1–C2 domains (the mouse counterpart of the E334K mutation in human cMyBP-C) also demonstrated a decrease in actin binding affinity [38].

The E334K substitution is located within the tri-helix bundle, a highly conserved region, and plays crucial roles across different muscle types, including actin binding. This mutation alters the protein’s electrical charges and local conformation, thereby weakening its affinity for actin [39]. However, the effects of cMyBP-C mutations on actin-binding affinity remain contradictory. For example, the D389V substitution was found not to affect cMyBP-C’s binding to actin filaments [40].

Earlier studies suggested that the cMyBP-C C2 domain did not serve as an attachment site on thin filaments. Instead, it seems to function as a brake, exerting a set counterforce to cardiac contraction [41]. These findings underscore how HCM mutation can impact the interaction of cMyBP-C with actin. Evidence from human C0–C2 fragments has shown that HCM mutations can variably affect actin binding. For instance, the L352P substitution enhances actin binding, whereas the R282W substitution disrupts phosphorylation, resulting in altered actin-binding affinity [42]. These results emphasize the importance of understanding the molecular changes accompanying these cMyBP-C mutations and their impact on protein structure and function [43].

The structural and functional alterations caused by the E334K substitution align with its association with haploinsufficiency and disrupted protein degradation pathways [17]. This mutation compromises cMyBP-C stability, leading to decreased protein levels and triggering hypertrophic signaling, a hallmark of HCM. Additionally, this substitution interferes with the ubiquitin–proteasome system, exacerbating protein degradation, cellular stress, and apoptosis. Furthermore, irregularities in UPS-related pathways could hinder Ca^2+^ ion regulation in cardiac muscle cells, potentially causing abnormal heart rhythms or contractile dysfunction [44,45]. In contrast, the milder effects of S236G substitution suggest it may represent a benign polymorphism but could still contribute to pathological hypertrophy when combined with sustained cardiac stress or other genetic factors.

The varying degree of severity of the S236G and E334K mutation can be partly attributed to their distinct positioning within the cMyBP-C protein structure. Residue S236 is located in a less conserved region, suggesting potentially milder effects on cMyBP-C function compared to E334, which resides in a highly conserved tri-helix bundle within the protein’s regulatory domain [43]. This distinctive region is crucial as it harbors key phosphorylation sites, underscoring the functional significance of the E334K mutation [37]. Unlike p.S236G, which exhibits thermodynamic stability and Tm values comparable to the wild-type protein, inducing only minor structural alterations without affecting protein-binding sites, the p.E334K variant showed a more severe phenotype. Previous studies demonstrated that this variant had lower expression levels and rapid degradation due to a shorter half-life, and elevated polyubiquitination [17,46]. Moreover, the p.E334K substitution reduces cellular 20S proteasome activity, increases the proapoptotic/antiapoptotic protein ratio, and enhances apoptosis in neonatal rat cardiomyocytes [46]. These reports demonstrated that the p.E334K mutation disrupts cMyBP-C stability, likely through the ubiquitin–proteasome system, contributing to cardiac dysfunction in HCM patients. While the p.S236G mutation seems to have milder functional implications, the pathological outcomes in both mutations require further investigation to clarify their roles in disease progression.

Importantly, HCM associated with these mutations may initially manifest as a reversible type of hypertrophy, akin to the physiological cardiac adaptation observed in high-performance athletes [47]. Indeed, during vigorous physical activity, the heart has the capacity to respond to stress by increasing muscle mass, a process referred to as cardiac hypertrophy. At early stages, cardiac hypertrophy can be linked to normal or enhanced cardiac function, recognized as adaptive or physiological hypertrophy [48]. The absence of other clinical symptoms among individuals carrying the p.S236G mutations suggests that this substitution may represent a benign polymorphism. Nevertheless, under sustained stress, this adaptive or physiological hypertrophy can progress into pathological hypertrophy, which is connected to impaired contractile function [48]. Typically, pathological hypertrophy manifests in the presence of cardiovascular conditions, potentially leading to the onset of heart failure [49,50]. Pathological hypertrophy is often characterized by elevated levels of circulating hormones, loss of cardiomyocytes, and compromised systolic and diastolic functions [51]. Vigorous physical training can induce a physiological adjustment within the cardiovascular system, but it also carries the risk of exacerbating heart-related complications such as arrhythmias, myocardial infarction, aortic dissection, and sudden cardiac arrest [20].

Previous research has established a direct correlation between mutations in the *MYBPC3* gene and cardiac dysfunction, resulting in hypertrophic cardiomyopathy. Da’as et al. found that *MYBPC3*-knockout zebrafish showed significant morphological changes in the heart during larval phases, including abnormal cardiac growth and dysfunction [52]. cMyBP-C demonstrates contradictory effects on the susceptibility to cardiovascular conditions. While certain genetic variations, like p.S236G, can enhance elite endurance and heart function, others, such as p.E334K, are linked to heart abnormalities [3]. p.S236G and p.E334K mutations directly affect the levels and function of cMyBP-C, but they also have an indirect impact on other cellular mechanisms relevant to normal cardiac activity. Moreover, it has been reported that the presence of a single mutation can lead to mild or severe symptoms and eventual heart failure, whereas patients expressing multiple cMyBP-C variants may present with typical HCM, characterized by diverse clinical symptoms in terms of disease severity, onset, and prognosis [37].

Our results demonstrate that discrete mutations of *MYBPC3* have distinct effects on the structure and function of cMyBP-C, with p.E334K mutant showing an increased thermal stability and decreasing the actin-binding affinity, whereas p.S236G exhibits stability similar to the wild-type, which may be linked to reversible hypertrophy observed in populations. These findings highlight the nuanced role of *MYBPC3* mutations in cardiac health, ranging from short-term functional benefits to pathological consequences. While it is currently challenging to perform genetic testing on all patients with HCM, assessing *MYBPC3* mutations is especially useful in certain cases. Genetic testing is valuable for patients with a family history of HCM or sudden cardiac death, those with early onset or severe forms of the disease, and those exhibiting atypical clinical features. Furthermore, identifying pathogenic variants allows for cascade screening in family members. Consequently, targeted genetic testing in this subset of HCM patients can provide important clinical and prognostic information. While our current findings provide deep insights into the structural and functional impacts of the p.S236G and p.E334K mutations, further in vivo studies using animal models, such as zebrafish and transgenic mice, are necessary to validate their physiological relevance and to deepen our understanding of genotype-phenotype relationships in HCM.

For example, a transgenic mouse model (p.E330K-Tg) demonstrated that this substitution led to a smaller diastolic left ventricular internal dimension [43]. Zebrafish offer unique advantages for studying the impact of such mutations on cardiac anatomy and physiology. Their transparent embryos and adults enable real-time visualization of heart development and function, while their low cost and high-throughput capacity allow for extensive experimental trials, making them invaluable tools for advancing HCM research. Advances in genomic technologies will support these efforts, enabling more thorough investigations into the genetic underpinnings of cardiomyopathy and potential therapeutic avenues.

## 5. Conclusions

Our study provides valuable insights into the structural and functional impact of two *MyBPC3* mutations using a combination of in silico modelling and experimental validation. We demonstrated that both mutations induce distinct changes in protein structure, stability, and actin-binding affinity. Notably, the p.E334K mutation showed a more pronounced effect on the protein folding, leading to significantly reduced actin interaction, and compromising protein degradation pathways, hallmarks linked to pathological hypertrophy in HCM disease. However, p.S236G exhibited milder structural changes and may represent a benign polymorphism, although it could still lead to the progression of disease under sustained physical stress.

Our findings highlight the importance of the effects of *MYBPC3* mutations, underscoring the variety of phenotypic variability and potential for both adaptive and pathological cardiac remodeling. Despite the fact that genetic screening remains challenging, due to its high cost, targeted testing, especially in high-risk patients, can offer significant clinical value, particularly for early screening and intervention. Further investigations using in vivo models, such as zebrafish and transgenic mice, are crucial to elucidate the genotype-phenotype correlations and underlying pathological mechanisms in HCM disease. In vivo studies will be instrumental in advancing our understanding of mutation-specific outcomes and in paving the way toward precision medicine approaches for the management of inherited cardiomyopathy.

## Figures and Tables

**Table 1:** Predicted pathogenicity and structural impact of missense mutations in cMyBP-C.

Protein	Mutation	SIFT	Polyphen2		DDmut	
cMyBP-C (Q14896)	p.S236G	Tolerated	Benign	Score = 0.001 sensitivity: 0.99 specificity: 0.15	Destabilizing	
					Predicted Δ ΔG = −0.39 kcal/mol	b-factor = −0.12
	p.E334K	Tolerated	Possibly damaging	score = 0.874 sensitivity: 0.83 specificity: 0.93	Stabilizing	
					Predicted Δ ΔG = 0.02 kcal/mol	b-factor = −0.33

**Table 2:** Secondary structure elements for all cMyBP-C proteins at 25 °C.

Protein	α-Helix	β-Sheet		β-Turn	Random Coil
		Antiparallel	Parallel		
**WT**	17.2%	13.4%	16.5%	16.2%	38.5%
**S236G**	17.9%	12.5%	15.7%	17.9%	37.6%
**E334K**	16.7%	13.7%	16.9%	15.3%	39.6%

**Table 3:** Summary of the thermodynamic parameters for the thermal unfolding of C0–C2 domains of cMyBP-C^WT^, cMyBP-C^S236G^, and cMyBP-C^E334K^ as monitored by DSC.

Protein Sample	ΔH_unf_ (kJ/mol)	T_m_ (°C)
WT	432 ± 11	54.7 ± 0.1
S236G	408 ± 5	54.2 ± 0.1
E334K	375 ± 7	58.4 ± 0.1

**Table 4:** Thermodynamic parameters calculated from chemical denaturation experiments for C0–C2 domain fragments of cMyBP-C^WT^, cMyBP-C^S236G^, and cMyBP-C^E334K^.

Protein Sample	m (kcal mol^−1^ M^−1^)	D^50%^ (M)	ΔG_DN_ (kcal/mol)
WT	2.38 ± 0.37	1.57 ± 0.06	3.74 ± 0.37
S236G	1.99 ± 0.18	1.65 ± 0.04	3.28 ± 0.30
E334K	2.64 ± 0.15	1.60 ± 0.02	4.22 ± 0.21

## Data Availability

Data is contained within the article.
